# A critical examination of a newly proposed interhemispheric teleconnection to Southwestern US winter precipitation

**DOI:** 10.1038/s41467-019-10528-y

**Published:** 2019-06-19

**Authors:** Peter B. Gibson, Duane E. Waliser, Michael J. DeFlorio

**Affiliations:** 1 Jet Propulsion Laboratory, California Institute of Technology, Pasadena, CA 91011 USA; 2 Center for Western Weather and Water Extremes, Scripps Institution of Oceanography, University of California, San Diego, CA 92093 USA

**Keywords:** Climate sciences, Atmospheric dynamics

**Arising from** A. Mamalakis et al. *Nature* 10.1038/s41467-018-04722-7 (2018)

In their recent study^1^, (hereafter M18) propose a new interhemispheric teleconnection linking late austral winter sea surface temperature (SST) anomalies in the New Zealand region to Southwestern US winter precipitation. They propose that warm SST anomalies propagate from the New Zealand region into the Northern Hemisphere through an atmospheric bridge mechanism (from approximately July to October) which in subsequent months (from November to March) acts to alter properties of the jet stream that influence Southwestern US winter precipitation variability. However, in this correspondence, after accounting for non-stationarity, memory and shared variability with other well-known modes of internal variability, we show a large reduction in correlation strength between SST anomalies in regions key to the proposed mechanism. Other important claims in M18 regarding the physical mechanisms involved, whereby SST anomalies in the New Zealand region enhance remote adiabatic warming and reduce cloud cover in key regions, are not robustly supported when examined in reanalysis and satellite data.

A critical aspect of the newly proposed teleconnection in M18 depends on SSTs in the New Zealand (NZI) region leading, and being a source of predictability for, western tropical Pacific SSTs in a region east of the Philippines (EPH). To reduce the likelihood of spurious correlation when searching for new teleconnections, it is crucial that any well-known confounders (i.e. other variables or processes that jointly influence SSTs in both regions) are given careful consideration. First, reasonably large trends in SSTs are known to exist in both of these regions over the time period considered in M18 (e.g. see ref. ^2^) (see also Supplementary Fig. 1). Second, the El Niño-Southern Oscillation (ENSO) and related oscillations, including the Southern Oscillation Index (SOI), are well known to influence SST anomalies and atmospheric circulation in both of these regions^3,4^ (see also Supplementary Fig. 1). Another important consideration when assessing predictability lies within the Granger causality paradigm^5,6^): that causation depends on the predictive strength of the independent variable (here SSTs in NZI) significantly exceeding the predictive strength of past values (i.e. memory) of the dependent variable alone (here SSTs in EPH). Lastly, it is well established that simultaneously conducting multiple hypothesis tests ensures a higher standard (i.e. lower *p*-values) for rejecting local null hypotheses^7^. The influence of each of these considerations (non-stationarity, memory, other confounders and significance testing) is examined in detail below.

The correlation strength between SSTs in NZI and EPH as a function of NZI lead time in months over the period 1982–2015 is shown in Fig. 1 (Fig. 1a is equivalent to Fig. 7 in M18). As discussed in M18, the strongest of these correlations (*r* = 0.85) occurs for NZI September SST anomalies leading EPH by 3–4 months and with other months/lags also displaying high correlations (*r* > 0.7) related to the atmospheric bridging mechanism proposed in M18 (i.e. NZI July to October SST anomalies leading EPH by 1–5 months). When the anomalies from both timeseries are linearly detrended before computing the correlation (Fig. 1b) the correlation strength during these important months/lags is reduced somewhat to the range of *r* = 0.52–0.76 but remains strongly statistically significant. However, EPH SST anomalies (after detrending) also display considerable memory (Fig. 1c) and in many months/lags display greater correlation strength than NZI as a predictor (comparing Fig. 1c with 1b). Furthermore, SOI is found to be very well correlated with EPH over the same months/lags of interest (comparing Fig. 1d with 1b) highlighting an important source of predictability for EPH SST anomalies that is also well correlated with SST anomalies in the NZI region (Supplementary Fig. 1).

**Fig. 1:**
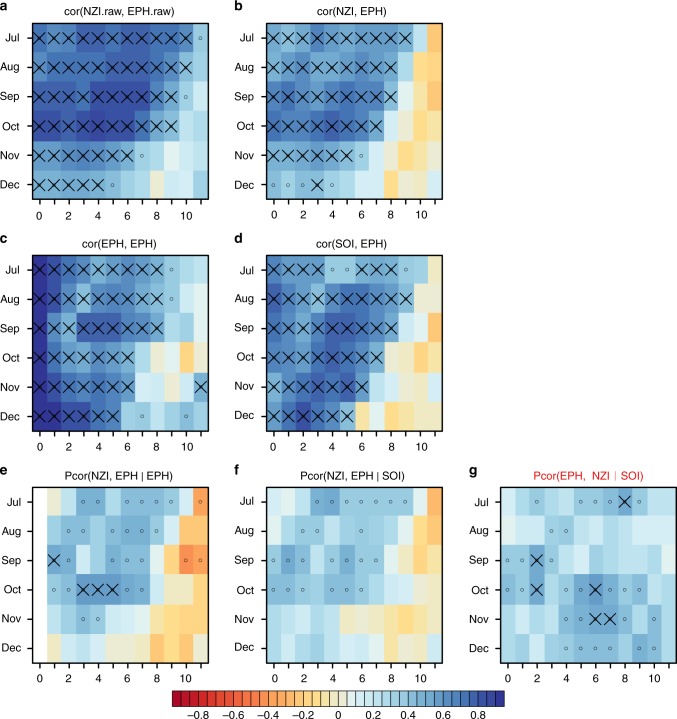
Time-lagged Pearson correlations where *y*-axis refers to month of predictor and *x*-axis refers to the number of months that the predictand is lagged forward. SST anomalies are calculated relative to month-of-year climatology. **a** Correlation between raw NZI SST anomalies leading raw EPH SST anomalies without detrending, note in all other panels time-series have been linearly detrended prior to correlation; **b** correlation of NZI leading EPH; **c** correlation of EPH with EPH showing memory in EPH SST anomalies; **d** correlation of SOI leading EPH; **e** partial correlation of NZI leading EPH after controlling for EPH memory; **f** partial correlation of NZI leading EPH after controlling for SOI; **g** direction is reversed from **f** i.e. the partial correlation of EPH leading NZI after controlling for SOI. In all panels black circles indicate local significance at *p* < 0.05, black crosses indicate significance at *p* < 0.05 after controlling the false discovery rate (FDR) under multiple hypothesis testing. SST data is from JRA COBE-SST2 covering the period 1982–2015

To more directly diagnose the relative influence of SOI and EPH memory in these associations, we present results of partial correlation analysis (e.g. ref. ^8,9^). When the influence from EPH memory is accounted for through partial correlation (Fig. 1e), the association between NZI leading EPH is substantially reduced in all months. Over key month/lags (i.e. NZI July to October SST anomalies leading EPH by 1–5 months) the correlation strength has reduced from *r* = 0.7–0.85 to *r* = 0.15–0.58 indicating that a considerable amount of the original association can be explained by EPH memory alone. When the influence from SOI is accounted for through partial correlation (Fig. 1f) we find that none of the NZI leading EPH correlations remain statistically significant (after accounting for multiple hypothesis testing) and the correlation strength over key month/lags has reduced considerably from *r* = 0.7–0.85 to *r* = 0.22–0.51. We also found that when the proposed causal pathway is analyzed in the reverse direction (i.e. predicting NZI SST anomalies from previous EPH SST anomalies and controlling for SOI through partial correlation, Fig. 1g) the strength of association is often larger (compared to Fig. 1f) and now displays statistical significance in some months/lags. In summary, these results indicate that the strong lagged associations for NZI leading EPH SST anomalies reported in M18 are inflated by already well-known processes and variables (not accounted for in M18) that jointly influence SST anomalies in these regions.

M18 suggested that the apparent association between NZI and EPH SSTs over the period 1982–2015 (but not before 1982) may be due to recent tropical expansion under anthropogenic forcing or due to poor data quality prior to 1982 masking the true correlation strength. To shed light on these considerations we analyzed multiple runs from the fully coupled CESM LENS historical experiment (see ref. ^10^. for details). Each run in the ensemble has the same historical forcings and parametrizations but slightly perturbed initial conditions. We found that many runs (e.g. Supplementary Fig. 2) display very weak lagged correlations (e.g. *r* = 0.2) over relevant months/lags but can strengthen (*r* > 0.5) and become strongly statistically significant in selected model runs (e.g. Supplementary Fig. 3). Since these runs differ only in terms of initial conditions, this suggests that any apparent lagged correlations found between these regions are not likely a consistent or emerging feature of the climate system, but instead occasionally arise due to stochastic internal climate variability.

Lastly, we examine the proposed physical mechanism in M18 detailing how SST anomalies in the NZI region might propagate north to the EPH region. According to M18, this occurs when warm SST anomalies in the NZI region are sufficient in magnitude to induce an anomalous Hadley circulation resulting in descending motion over EPH, suppressed cloud formation and positive SST anomalies. By analyzing ERA-Interim vertical velocity at the 500-hPa level during the 5 warmest NZI years (as in Fig. 6 of MH18), we show that strong ascending motion remains apparent over the EPH region with no consistent reduction in strength of ascending motion (Fig. 2a, d, g). There is also no consistent reduction in total cloud cover (combined day and night) over the EPH region compared to climatology (Fig. 2b, e, h); instead total cloud cover slightly increased during NZI warm years. This result is supported in CERES EBAF satellite data (Fig. 2c, f, i) in the years of available data (2000-present).

**Fig. 2:**
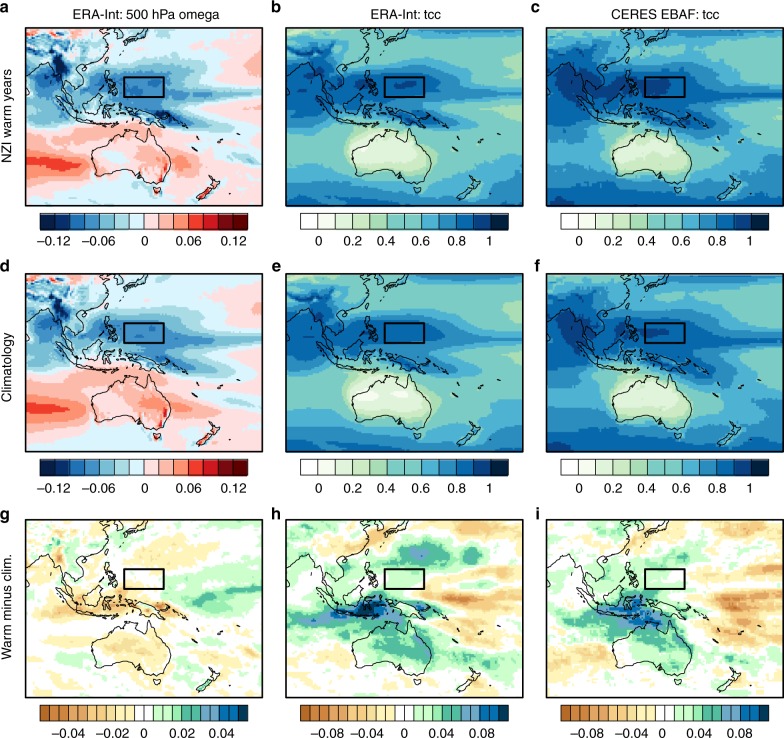
July–September ERA-Interim composites during the 5 warmest NZI years (based on ranked July–September SST anomalies) compared to climatology (1979–2016). **a**, **d**, **g** 500-hPa omega velocity (Pa/s, negative values indicate ascending motion); **b**, **e**, **h** total cloud cover (tcc). **c**, **f**, **i** are tcc from CERES EBAF satellite data (years 2000–2016). Due to the CERES EBAF data beginning in 2000, one less year was used to construct the composite in **c**. Almost identical results were found when NZI warm years and associated composites were instead calculated for September–November (not shown). The EPH region is indicated by a black box

In summary, a critical underpinning for the proposed teleconnection in M18 depends on an atmospheric bridge mechanism whereby SST anomalies propagate from NZI to EPH beginning in austral winter. However, we have shown that after accounting for significant non-stationarity, memory, multiple hypothesis testing, and other confounders there is currently little observational evidence that NZI SST anomalies offer a unique source of predictability for EPH SST anomalies. Second, using the CESM LENS model ensemble, we have illustrated that apparent lagged associations of the magnitude reported in M18 can occasionally arise from stochastic internal variability. Lastly, other mechanistic claims in M18 for how SST anomalies propagate between these key regions, are not robustly supported in reanalysis or satellite data. In combination, we argue that the physical basis for the proposed new teleconnection, derived solely from observations in M18, remains unclear. Without this, there is no indication that the proposed NZI index will be a useful source of predictability in future years (i.e. out-of-sample) for Southwestern US winter precipitation.

## Supplementary information

Supplementary Information

## Data Availability

All data used in this study are freely availability. SOI data used were from: ftp://ftp.bom.gov.au/anon/home/ncc/www/sco/soi/soiplaintext.html provided by the Australian Bureau of Meteorology. Observational SST data were from JRA COBE-SST2 from: https://www.esrl.noaa.gov/psd/data/gridded/data.cobe2.html. CESM LENS SST data were obtained from http://www.cesm.ucar.edu/projects/community-projects/LENS/data-sets.html. ERA-Interim omega and tcc data were from: http://apps.ecmwf.int/datasets/data/interim-full-daily/levtype=sfc. CERES EBAF v4 tcc data were from 10.5067/TERRA+AQUA/CERES/EBAF-TOA_L3B.004.0.
