# Supplementary material for: A critical examination of a newly proposed interhemispheric teleconnection to Southwestern US winter precipitation
Source: Nat Commun. 2019 Jun 19;10:2687. doi: 10.1038/s41467-019-10528-y (PMC6584695; doi:10.1038/s41467-019-10528-y)
Supplement: Supplementary file 1 — Supplementary Information [file 41467_2019_10528_MOESM1_ESM.pdf]

SUPPLEMENTARY MATERIAL

A critical examination of a newly proposed interhemispheric teleconnection to Southwestern US winter precipitation

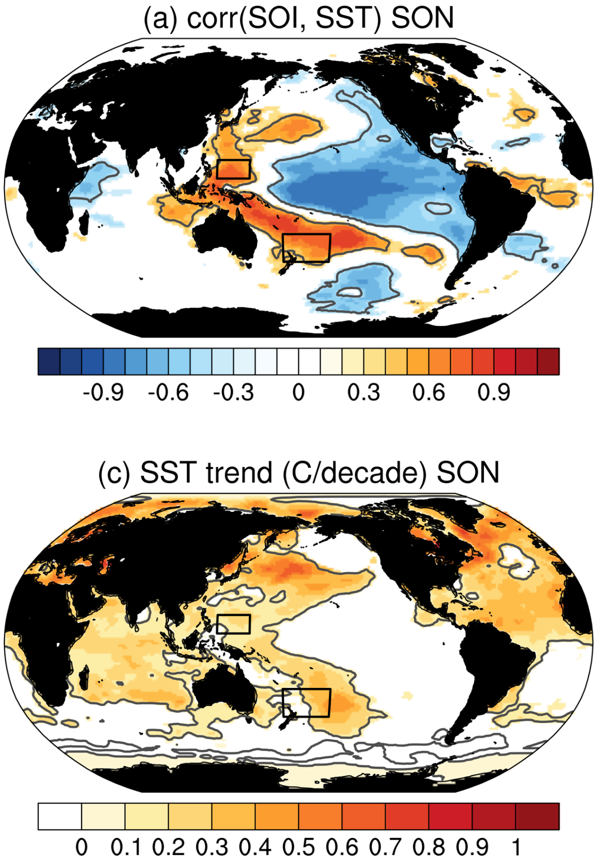

Supplementary Figure 1: (a) detrended concurrent correlation between SOI and SST anomalies for September-November (SON); (b) detrended concurrent correlation between SOI and SST anomalies for July-August (JAS); (c) linear trend in SST anomalies for SON; (d) linear trend in SST anomalies for JAS. Only values with local significance of $p<0.05$ are shown. Grey contour further indicates significance at $p<0.05$ after controlling the false discovery rate (FDR). Boxes indicate the NZI and EPH regions. SST data is from JRA COBE-SST2 over the period 1982-2015.

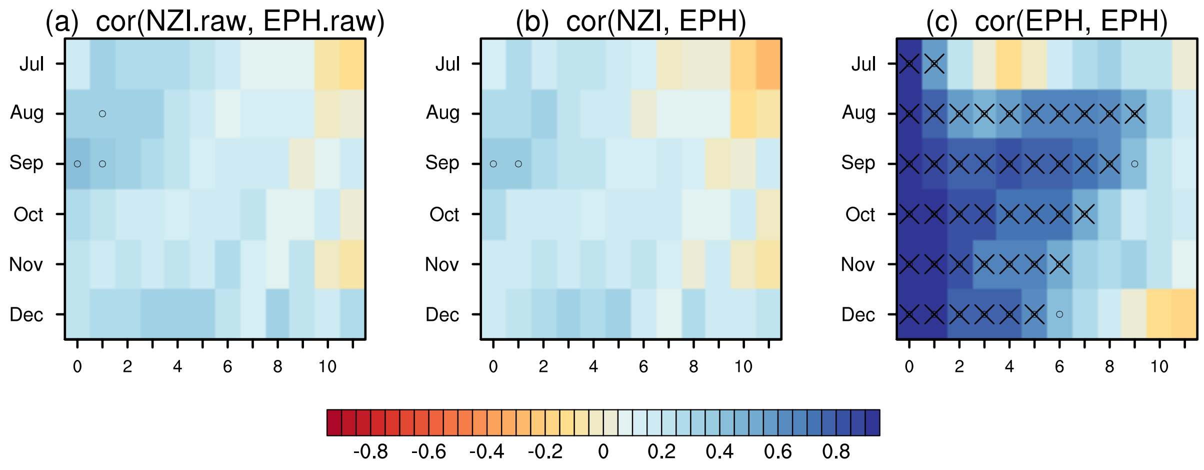

Supplementary Figure 2: As in Figure 1a-c of main paper but here for ‘run 019’ of the CESM LENS model ensemble for the period 1972-2005.

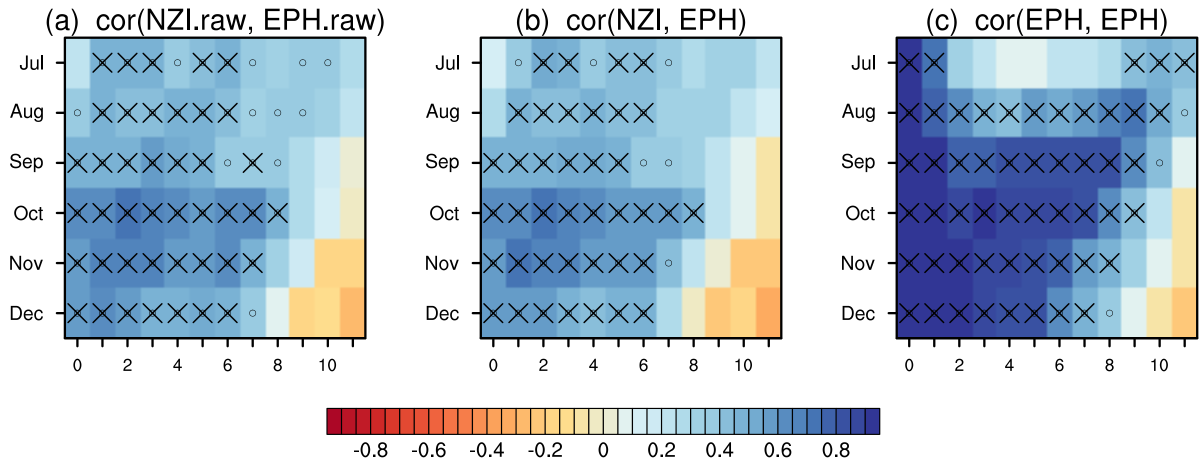

Supplementary Figure 3: As in Figure 1a-c of main paper but here for ‘run 033’ of the CESM LENS model ensemble for the period 1972-2005.
